# Lutein administration during gestation protects morphine withdrawal-induced on reflexive motor behavior in mice offspring's

**DOI:** 10.1016/j.heliyon.2025.e42394

**Published:** 2025-01-31

**Authors:** Sahand Sariaslani, Shahin Hassanpour, Bita Vazir

**Affiliations:** a Faculty of Veterinary Medicine, Science and Research Branch, Islamic Azad University, Tehran, Iran; b Division of Physiology, Department of Basic Sciences, Faculty of Veterinary Medicine, Science and Research Branch, Islamic Azad University, Tehran, Iran

**Keywords:** Lutein, Morphine withdrawal, Reflexive motor behavior, Offspring's, Mice

## Abstract

Exposure to morphine during gestation period has vital effect on infants’ neurodevelopmental growth pattern. Lutein shows protective qualities regarding neurodegeneration and the development of the brain. This study aimed to investigate effect of parental exposure to lutein on adverse effect of the morphine withdrawal syndrome on reflexive motor behavior in mice offspring. Fourteen male mice and 56 adult female mice were randomly assigned to seven groups: the control group containing male and female mice that refrained from morphine; group 2 was morphine-abstinent male mice and female mice with no prior drug exposure; group 3 including morphine-abstinent female mice and male mice without drug experience; and group 4 of drug-naïve male and female mice. Groups 5–7 were similar to groups 2–4, but drug-naïve subjects received injections of lutein (10 mg/kg). Following delivery, offspring from each group were chosen to assess behavior and reflexive motor behaviors. Also, blood samples were collected to measure serum antioxidant activity. Based on the findings, reflexive motor behaviors significantly decreased following prenatal exposure to morphine (P < 0.05), however, no significant difference was seen between morphine exposed male with male and female morphine exposed mice (P > 0.05). Lutein administration had no effect on reflexive motor behaviors in male morphine exposed group (P > 0.05) but lutein administration significantly decreased adverse effect of the morphine on reflexive motor behaviors in female exposed group (P < 0.05). No significant difference was seen between male and females received morphine (P > 0.05). Lutein administration decreased serum malondialdehyde (MDA) production and increased superoxide dismutase (SOD), glutathione peroxidase (GPx) and total antioxidant status (TAS) levels in morphine exposed mice. It seems, maternal exposure to lutein had protective role on adverse effect of the morphine on reflexive motor behaviors in offspring.

## 1 Introduction

Opioids are often used to relieve pain and are widely utilized as a psychoactive substance [1]. The long-term use of opiates or their abuse leads to the emergence of physical and psychological dependence. Substance addiction is a chronic and repeating condition viewed as an essential pursuit for drugs, with the emergence of adverse feelings in the lack of these substances [2]. In the withdrawal phase, anxiety intensifies, and negative reinforcement occurs, sometimes manifesting as a clinically dangerous condition [3]. Exposure to morphine in the neonatal period causes lasting changes in pain pathways. Exposure to morphine and neurodevelopmental results in infants born extremely preterm. Additionally, in neonatal rats, inadequate levels of morphine lead to hyperalgesia during adolescence [4]. The prevalence of prenatal opioid exposure continues to rise, as evidenced by the 82 % increase in cases of neonatal opioid withdrawal syndrome [5]. The young of adult rats that were exposed to methadone in the later phases of pregnancy and during nursing exhibited specific deficits in executive function related to cognitive flexibility, though this was not assessed by sex [6]. Evidence suggests that exposing adult male and female rats to morphine leads to behavioral and structural changes in their offspring [7]. In addition, a dysfunction of corticotropin-releasing factor (CRF) was noted in the male offspring of female rats that had been exposed to morphine during their adolescence [8].

Lutein, a carotenoid found in green and leafy plants, can cross both the blood-retina barrier and the blood-brain barrier, enabling its delivery to different brain areas including the cortex, cerebellum, striatum, and hippocampus [9]. Lutein has the capacity to reduce lipid peroxidation and MDA production, while also restoring the levels of GPx, SOD, and CAT in the brain [24]. The use of lutein shows protective effects regarding neurodegeneration and brain development, due to its multiple mechanisms of action that include anti-inflammatory, antioxidant, and anti-apoptotic functions [10]. Moreover, it has been demonstrated that lutein supplementation can enhance the levels of brain-derived neurotrophic factor.

There is evidence supporting the protective effect of carotenoids against withdrawal syndrome. Carotenoids enhance the expression of BDNF and CREB genes in the ventral tegmental area of rats treated with morphine [11]. Additionally, carotenoids interact with the opioid system to alleviate withdrawal symptoms [23]. The protective function of carotenoids during ketamine withdrawal could be connected to their interaction with the cellular antioxidant defense system, as carotenoids reduce MDA production and enhance levels of SOD, GPx, and catalase [12]. According to literature, the beneficial effects of carotenoids on morphine withdrawal syndrome are well established, yet there is no record concerning the impact of lutein. Therefore, this research sought to examine the impact of parental exposure to lutein on the negative consequences of morphine withdrawal syndrome on the reflexive motor behavior of offspring and the levels of MDA, GPx, SOD, and TAS in the brain tissue of both mothers and their young.

## 2 Material and methods

### 2.1 Animals

Fourteen male and 56 virgins female NMRI mice (weighing 28–30 g and aged 8–10 weeks), were obtained from the Pasture Institute (Tehran, Iran). They were housed in standard plastic cages under laboratory conditions, with a temperature of 22 ± 2 °C and a 12-h light/dark cycle. The mice had access to standard chow pellets and fresh water. Next, they were randomly assigned to seven groups (with two males and eight females in each). Each morning, the female mice underwent evaluation to determine the presence of sperm or a vaginal plug, which indicated the possibility of pregnancy.

The first group served as the control group, consisting of male and female mice that did not receive morphine treatment. In the second group, female mice underwent morphine exposure for 21 days, whereas the male mice stayed abstinent from morphine. In the third group, male mice were exposed to morphine for 21 days, while the females remained morphine-abstinent. The fourth group both male and female mice exposed to morphine for 21 days. Groups of five female mice were exposed to morphine for 21 days, with the females being drug-naïve. The drug-naïve female mice were administered lutein (10 mg/kg) on gestational days 5, 8, 11, 14, and 17. In group six male mice were exposed to morphine for 21 days, with the males being drug-naïve. However, the females in this group were morphine-abstinent. The drug-naïve male mice were administered lutein at a dose of 10 mg/kg on days 5, 8, 11, 14, and 17. In group 7, both male and female mice were exposed to morphine for 21 days, with both genders being drug-naïve. Drug-naïve females were administered lutein (10 mg/kg) on days 5, 8, 11, 14, and 17days of gestation. Male mice were administered lutein (10 mg/kg) on the same days. The Animal Ethics Committee of the Science and Research Branch of Islamic Azad University, Tehran, Iran (IR.IAU.SRB.REC. 1402.171) granted approval for all experimental procedures. After the mice delivered, 20 male pups from every litter were chosen according to the distance from the anus to the vagina. These puppies were subsequently utilized to perform postnatal (PD) reflex and neonatal motor behavior assessments. To avoid problems associated with body warmth and hunger, the mice young were briefly kept apart from their mother for up to 15 min [13].

### 2.2 Drugs and antioxidant activity

Drugs including lutein (CAS No. 127-40-2, Sigma co. USA) and morphine hydrocholoride (Osel, Turkey) were purchased in this study. After completing all behavioral assessments, cranium dissection was performed and brain tissue of the MDA (Catalog No.: ZB-MDA-48A), SOD (Catalog No.: ZB-SOD-48A), GPx (Catalog No.: ZB-GPX-A48), and TAS (Catalog No.: ZB-TAS-48A) levels in both mothers and offspring were determined using assay kits.

### 2.3 Ambulation

Crawling behavior is noted in young mice up to postnatal day 5, after which they start walking between 5 and 10 days old. The walking assessment was conducted on 5-day-old offspring to utilize this transitional phase. The mice were situated in a clear chamber that provided visibility from above and all four sides. To promote walking, a soft nudge of the tail was used. The ambulation assessment employed a grading scale with four levels: a score of 0 represented no movement, a score of 1 signified crawling with uneven limb motion, a score of 2 indicated slow crawling with even limb motion, and a score of 3 signified fast crawling or walking. To reduce the impact of learning on the test outcomes, the experiment was conducted three times over a 3-min span [13].

### 2.4 Hind-limb foot angle

The study aimed to analyze the hind-limb foot angle in 8-day-old offspring (PD 8) to investigate changes in hind-limb posture during the developmental transition from crawling to ambulation. As the pups matured and started walking, their hind limbs shifted position to be located beneath their bodies. Consequently, the angle formed by the hind limbs during walking was observed to be smaller compared to that during crawling. To observe the motion of the pups, a simple enclosure with an unobstructed view, equipped with a video recorder, was used. The pups were encouraged to walk by gently touching their tails. The measurement of foot angle was performed using recorded videos, where a line was drawn from the end of the heel/shin to the tip of the middle digit. Foot angle measurements were limited to pups that executed a complete stride in a linear trajectory with their feet in a flat position on the ground. To minimize potential experimental errors, foot angle averages were calculated from three to five sets of measurements for each pup. The test results did not show any evidence of learning-related effects [13].

### 2.5 Surface righting

The research intended to examine the hind-limb foot angle in 8-day-old young (PD 8) to explore alterations in hind-limb stance throughout the developmental shift from crawling to walking. As the puppies grew and began to walk, their back legs adjusted to a position directly under their bodies. As a result, the angle created by the hind limbs while walking was noted to be lesser than when crawling. A straightforward enclosure with a clear view, fitted with a video recorder, was utilized to watch the pups' movement. The puppies were motivated to move by softly tapping their tails. The foot angle was measured by utilizing recorded videos, in which a line was traced from the heel/shin's end to the middle toe's tip. Foot angle measurements were restricted to pups that performed a full stride in a straight line with their feet flat against the ground. To reduce possible experimental errors, average foot angles were computed from three to five measurement sets for each pup. The test outcomes revealed no signs of effects related to learning [13].

### 2.6 Grip strength

This evaluation measured grip strength in puppies aged PD 5–15. Nonetheless, to guarantee accuracy and uniformity, the experiment was carried out on PD 8 progeny. The aim of the research was to assess how well animals can hold onto a screen and generate a measurement of grip strength. To assess the grasping capacity of all four limbs, a fiberglass screen measuring 16 × 18 was slowly turned from a horizontal to a vertical orientation. The hanging force, representing the strength needed to counteract gravity, was determined by the formula: [fall time (s) × weight (g)] [13].

### 2.7 Negative geotaxis

The negative geotaxis test, assessing the vestibular reaction to gravity, was performed on mice at postnatal day 8, though it is relevant for postnatal days 3–15 [14]. The mice were positioned on a wooden slope inclined at 45° and set free, while the duration for them to align themselves upwards was measured and noted [13].

### 2.8 Statistical analysis

The obtained data were analyzed using one-way analysis of variance (ANOVA) and presented as mean ± standard error (SE). For treatments showing significant differences, mean values were compared using the Tukey HSD test. Treatments with P-values less than 0.05 were considered to have significant differences.

## 3 Results

**Fig. 1:**
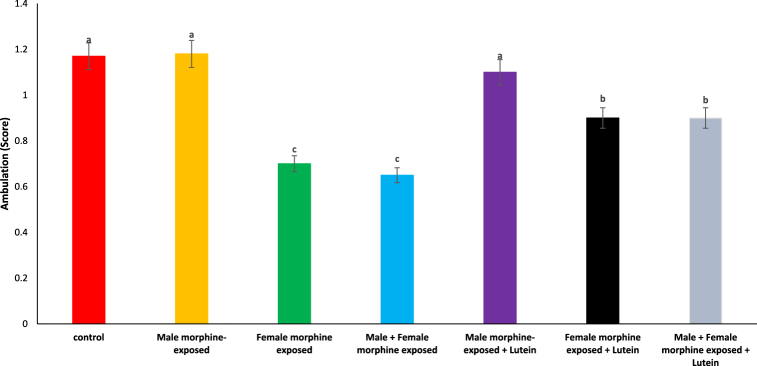
Effects of the Lutein administration during pregnancy in parental with morphine withdrawal on ambulation score in mice offspring's. Drug-naïve male injected with Lutein (10 mg/kg) on days 5, 8, 11, 14 and 17 days. Drug-naïve female injected with Lutein (10 mg/kg) on days 5, 8, 11, 14 and 17 days of gestation. Data are expressed as mean ± SE. There are significant differences between groups with different superscripts (a, b and c; P < 0.05).

Table 1 shows the effects of Lutein administration during pregnancy in parents undergoing morphine withdrawal, including comparisons and their significance. The impact of Lutein administration on the ambulation score in the offspring of mice is illustrated in Fig. 1. It was observed that there was no significant difference in ambulation scores between the male morphine-exposed group and the control group (P > 0.05). The ambulation score showed a significant reduction after prenatal morphine exposure [F (6, 48): 1.23, P < 0.05], but there was no significant difference observed between the male morphine-exposed group and the combined male and female morphine-exposed group (P > 0.05). The administration of lutein in the male morphine-exposed group did not influence the ambulation score (P > 0.05), whereas in the female exposed group, lutein administration significantly reduced the negative impact of morphine on the ambulation score [F (6, 48): 1.11, P < 0.05]. No significant difference was observed between males and females treated with morphine when compared to females exposed to morphine and lutein (P > 0.05). Maternal exposure to lutein appears to have a protective effect against the negative impacts of morphine withdrawal on their offspring, while exposure to either morphine or lutein showed no effects on the offspring.

**Table 1:** A summary table with comparisons and significance.

	Control	Male morphine-exposed	Female morphine exposed	Male + Female morphine exposed	Male morphine-exposed + Lutein	Female morphine exposed + Lutein	Male + Female morphine exposed + Lutein
**ambulation score**	–	–	↓	↓	–	↑	↑
**hindlimb foot angle**	–	–	↑	↑	–	↓	↓
**surface righting**	–	–	↓	↓	–	↑	↑
**hindlimb suspension**	–	–	↓	↓	–	↑	↑
**grip strength**	–	–	↓	↓	–	↑	↑
**front limb suspension**	–	–	↓	↓	–	↑	↑
**negative geotaxis**	–	–	↑	↑	–	↓	↓
**MDA**	–	–	↑	↑	–	↓	↓
**SOD**	–	–	↓	↓	–	↑	↑
**GPx**	–	–	↓	↓	–	↑	↑
**TAS**	–	–	↓	↓	–	↑	↑

-: no effect, ↓: decreased on negative effect, ↑: increased or positive effect.

**Fig. 2:**
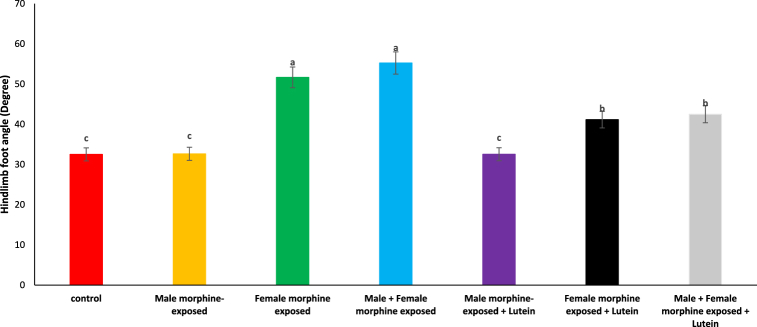
Effects of the Lutein administration during pregnancy in parental with morphine withdrawal on hindlimb foot angle in mice offspring's. Drug-naïve male injected with Lutein (10 mg/kg) on days 5, 8, 11, 14 and 17 days. Drug-naïve female injected with Lutein (10 mg/kg) on days 5, 8, 11, 14 and 17 days of gestation. Data are expressed as mean ± SE. There are significant differences between groups with different superscripts (a, b and c; P < 0.05).

As seen in Fig. 2, there was no significant difference on hindlimb foot angle degree in male morphine exposed group compared to control group (P > 0.05). Hindlimb foot angle degree significantly increased following prenatal exposure to morphine [F (6, 48): 12.26, P < 0.05], however, no significant difference was seen between male morphine-exposed group compared to male and female morphine-exposed group (P > 0.05). Lutein administration in male morphine exposed group had no effect on hindlimb foot angle degree (P > 0.05) but lutein administration in female exposed group significantly decreased adverse effect of the morphine on hindlimb foot angle degree [F (6, 48): 10.11, P < 0.05]. No significant difference was seen between male and females treated with morphine and compared to female exposed to morphine and lutein (P > 0.05). Maternal exposure to lutein appears to have positively influenced the negative impact of morphine withdrawal on their offspring, while exposure to either morphine or lutein did not affect their offspring's.

**Fig. 3:**
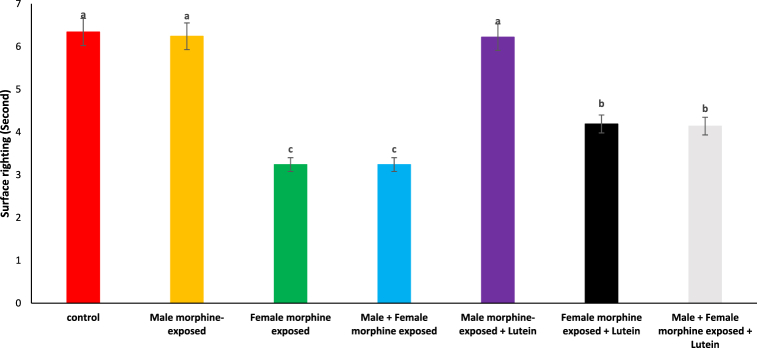
Effects of the Lutein administration during pregnancy in parental with morphine withdrawal on surface righting in mice offspring's. Drug-naïve male injected with Lutein (10 mg/kg) on days 5, 8, 11, 14 and 17 days. Drug-naïve female injected with Lutein (10 mg/kg) on days 5, 8, 11, 14 and 17 days of gestation. Data are expressed as mean ± SE. There are significant differences between groups with different superscripts (a, b and c; P < 0.05).

Based on Fig. 3, there was no significant difference on surface righting time in male morphine exposed group compared to control group (P > 0.05). Surface righting time significantly decreased following prenatal exposure to morphine [F (6, 48): 1.42, P < 0.05], however, no significant difference was seen between male morphine exposed group compared to male and female morphine exposed group (P > 0.05). Lutein administration in male morphine exposed group had no effect on surface righting time (P > 0.05) but lutein administration in female exposed group significantly decreased adverse effect of the morphine on surface righting time [F (6, 48): 1.61, P < 0.05]. No significant difference was seen between male and females treated with morphine and compared to female exposed to morphine and lutein (P > 0.05). It seems, maternal exposure to lutein had protective role on adverse effect of the morphine withdrawal on their offspring but expose to morphine or lutein had no effect on their offspring's.

**Fig. 4:**
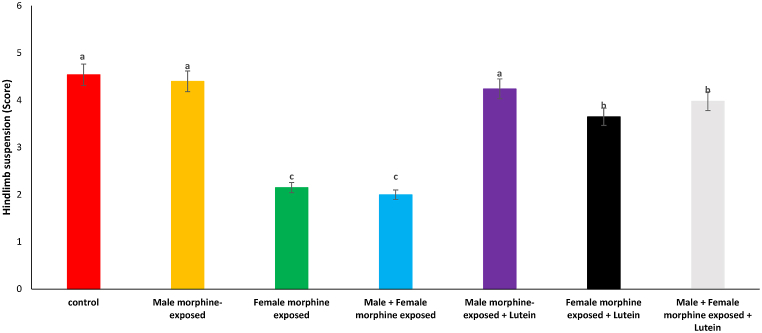
Effects of the Lutein administration during pregnancy in parental with morphine withdrawal on hindlimb suspension in mice offspring's. Drug-naïve male injected with Lutein (10 mg/kg) on days 5, 8, 11, 14 and 17 days. Drug-naïve female injected with Lutein (10 mg/kg) on days 5, 8, 11, 14 and 17 days of gestation. Data are expressed as mean ± SE. There are significant differences between groups with different superscripts (a, b and c; P < 0.05).

As shown on Fig. 4, there was no significant difference on hindlimb suspension score in male morphine exposed group compared to control group (P > 0.05). Hindlimb suspension score significantly decreased following prenatal exposure to morphine [F (6, 48): 1.36, P < 0.05], however, no significant difference was seen between male morphine exposed group compared to male and female morphine exposed group (P > 0.05). Lutein administration in male morphine exposed group had no effect on hindlimb suspension score (P > 0.05) but lutein administration in female exposed group significantly decreased adverse effect of the morphine on hindlimb suspension score [F (6, 48): 1.13, P < 0.05]. No significant difference was seen between male and females treated with morphine and compared to female exposed to morphine and lutein (P > 0.05). It seems, maternal exposure to lutein had beneficial effect on adverse effect of the morphine withdrawal on their offspring but expose to morphine or lutein had no effect on their offspring's.

**Fig. 5:**
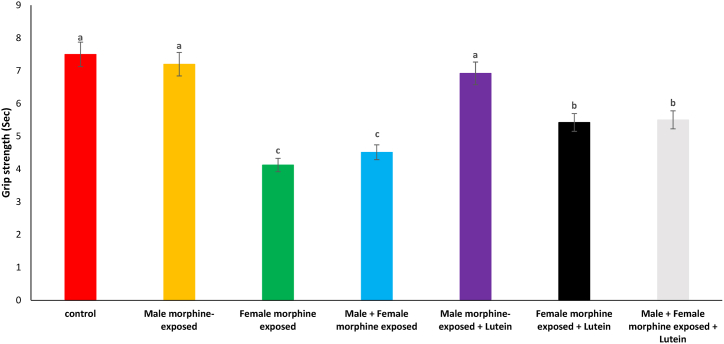
Effects of the Lutein administration during pregnancy in parental with morphine withdrawal on grip strength in mice offspring's. Drug-naïve male injected with Lutein (10 mg/kg) on days 5, 8, 11, 14 and 17 days. Drug-naïve female injected with Lutein (10 mg/kg) on days 5, 8, 11, 14 and 17 days of gestation. Data are expressed as mean ± SE. There are significant differences between groups with different superscripts (a, b and c; P < 0.05).

Effects of the Lutein administration during pregnancy in parental with morphine withdrawal on grip strength in mice offspring's is presented in Fig. 5. Based on results, there was no significant difference on grip strength in male morphine exposed group compared to control group (P > 0.05). Grip strength score significantly decreased following prenatal exposure to morphine [F (6, 48): 1.41, P < 0.05], however, no significant difference was seen between male morphine exposed group compared to male and female morphine exposed group (P > 0.05). Lutein administration in male morphine exposed group had no effect on grip strength (P > 0.05) but lutein administration in female exposed group significantly decreased adverse effect of the morphine on grip strength [F (6, 48): 1.20, P < 0.05]. No significant difference was seen between male and females treated with morphine and compared to female exposed to morphine and lutein (P > 0.05). It seems, maternal exposure to lutein had protective role on adverse effect of the morphine withdrawal on their offspring but expose to morphine or lutein had no effect on their offspring's.

**Fig. 6:**
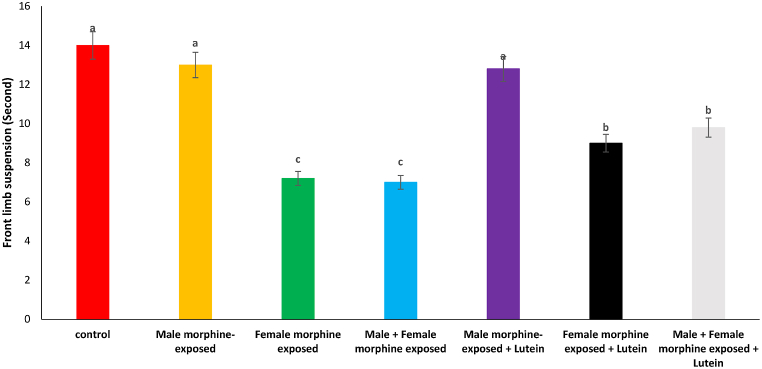
Effects of the Lutein administration during pregnancy in parental with morphine withdrawal on front limb suspension in mice offspring's. Drug-naïve male injected with Lutein (10 mg/kg) on days 5, 8, 11, 14 and 17 days. Drug-naïve female injected with Lutein (10 mg/kg) on days 5, 8, 11, 14 and 17 days of gestation. Data are expressed as mean ± SE. There are significant differences between groups with different superscripts (a, b and c; P < 0.05).

As seen in Fig. 6, there was no significant difference on front limb suspension time in male morphine exposed group compared to control group (P > 0.05). Front limb suspension time significantly decreased following prenatal exposure to morphine [F (6, 48): 2.03, P < 0.05], however, no significant difference was seen between male morphine exposed group compared to male and female morphine exposed group (P > 0.05). Lutein administration in male morphine exposed group had no effect on front limb suspension time (P > 0.05) but lutein administration in female exposed group significantly decreased adverse effect of the morphine on front limb suspension time [F (6, 48): 1.95, P < 0.05]. No significant difference was seen between male and females treated with morphine and compared to female exposed to morphine and lutein (P > 0.05). It seems, expose to morphine or lutein had no effect on front limb suspension time on their offspring's.

**Fig. 7:**
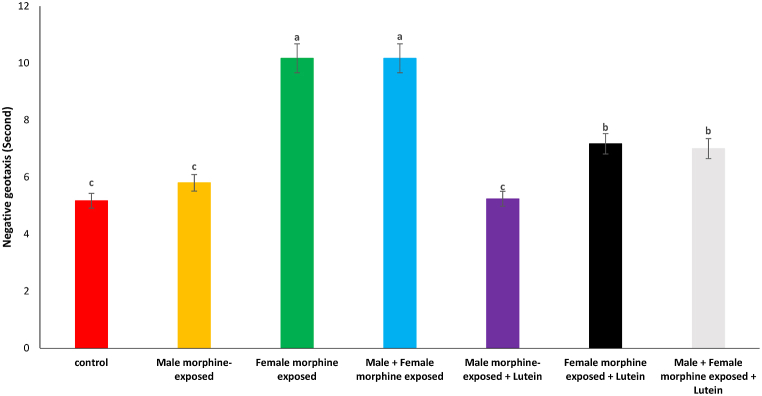
Effects of the Lutein administration during pregnancy in parental with morphine withdrawal on negative geotaxis in mice offspring's. Drug-naïve male injected with Lutein (10 mg/kg) on days 5, 8, 11, 14 and 17 days. Drug-naïve female injected with Lutein (10 mg/kg) on days 5, 8, 11, 14 and 17 days of gestation. Data are expressed as mean ± SE. There are significant differences between groups with different superscripts (a, b and c; P < 0.05).

According to Fig. 7, there was no significant difference on negative geotaxis in male morphine exposed group compared to control group (P > 0.05). Negative geotaxis significantly increased following prenatal exposure to morphine [F (6, 48): 1.61, P < 0.05], however, no significant difference was seen between male morphine exposed group compared to male and female morphine exposed group (P > 0.05). Lutein administration in male morphine exposed group had no effect on negative geotaxis (P > 0.05) but lutein administration in female exposed group significantly decreased adverse effect of the morphine on front limb suspension time [F (6, 48): 1.71, P < 0.05]. No significant difference was seen between male and females treated with morphine and compared to female exposed to morphine and lutein (P > 0.05). It seems, maternal exposure to lutein had protective role on adverse effect of the morphine withdrawal on their offspring but expose to morphine or lutein had no effect on their offspring's.

**Fig. 8:**
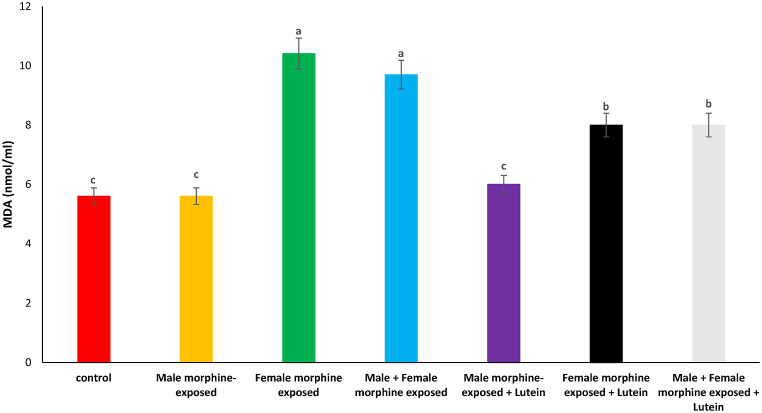
Effects of the Lutein administration during pregnancy in parental with morphine withdrawal on serum MDA production in mice offspring's. Drug-naïve male injected with Lutein (10 mg/kg) on days 5, 8, 11, 14 and 17 days. Drug-naïve female injected with Lutein (10 mg/kg) on days 5, 8, 11, 14 and 17 days of gestation. Data are expressed as mean ± SE. There are significant differences between groups with different superscripts (a, b and c; P < 0.05).

**Fig. 9:**
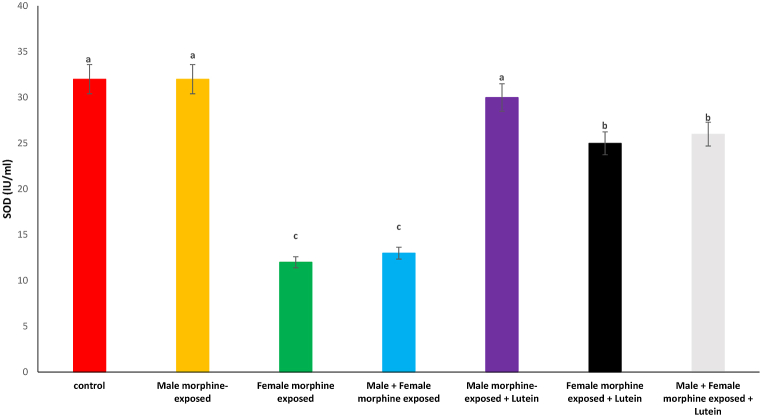
Effects of the Lutein administration during pregnancy in parental with morphine withdrawal on serum SOD levels in mice offspring's. Drug-naïve male injected with Lutein (10 mg/kg) on days 5, 8, 11, 14 and 17 days. Drug-naïve female injected with Lutein (10 mg/kg) on days 5, 8, 11, 14 and 17 days of gestation. Data are expressed as mean ± SE. There are significant differences between groups with different superscripts (a, b and c; P < 0.05).

**Fig. 10:**
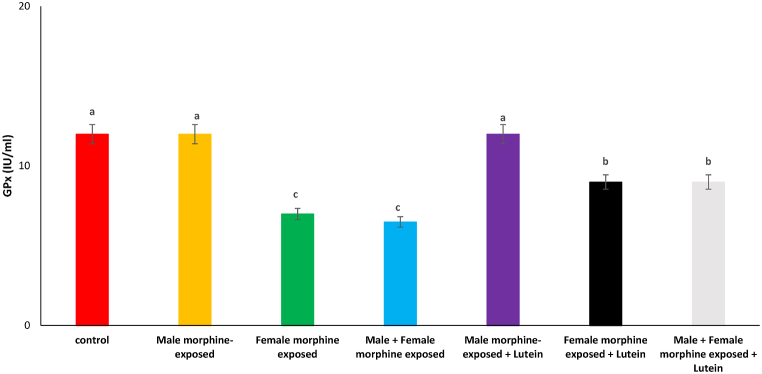
Effects of the Lutein administration during pregnancy in parental with morphine withdrawal on serum Gpx levels in mice offspring's. Drug-naïve male injected with Lutein (10 mg/kg) on days 5, 8, 11, 14 and 17 days. Drug-naïve female injected with Lutein (10 mg/kg) on days 5, 8, 11, 14 and 17 days of gestation. Data are expressed as mean ± SE. There are significant differences between groups with different superscripts (a, b and c; P < 0.05).

**Fig. 11:**
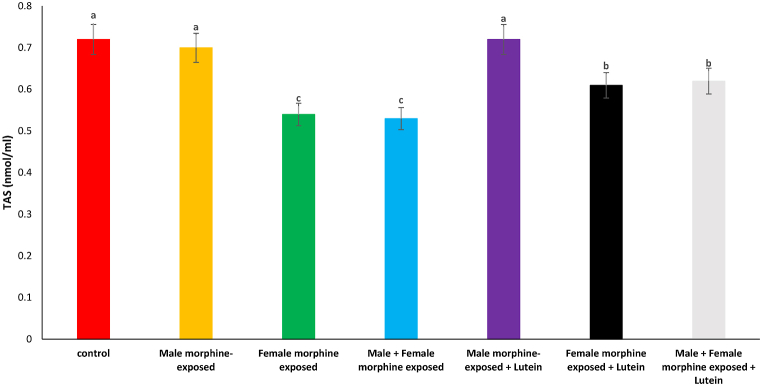
Effects of the Lutein administration during pregnancy in parental with morphine withdrawal on serum TAS levels in mice offspring's. Drug-naïve male injected with Lutein (10 mg/kg) on days 5, 8, 11, 14 and 17 days. Drug-naïve female injected with Lutein (10 mg/kg) on days 5, 8, 11, 14 and 17 days of gestation. Data are expressed as mean ± SE. There are significant differences between groups with different superscripts (a, b and c; P < 0.05).

Effects of the Lutein administration during pregnancy in parental with morphine withdrawal on serum MDA, SOD, GPx and TAS levels are shown in Fig. 8, Fig. 9, Fig. 10, Fig. 11. As seen, there was no significant difference on serum MDA, SOD, GPx and TAS levels in male morphine exposed group’ offspring compared to control group (P > 0.05). Serum MDA production increased with SOD, GPx and TAS levels decreased following prenatal exposure to morphine [F (6, 48): 1.31, P < 0.05], however, no significant difference was seen between male morphine exposed group compared to male and female morphine exposed group (P > 0.05). Lutein administration in male morphine exposed group had no effect on serum antioxidant levels (P > 0.05) but lutein administration in female exposed group significantly decreased adverse effect of the morphine on serum antioxidant levels [F (6, 48): 1.25, P < 0.05]. No significant difference was seen between male and females treated with morphine and compared to female exposed to morphine and lutein (P > 0.05). It seems, maternal exposure to lutein had protective role on adverse effect of the morphine withdrawal on serum antioxidant levels in offspring but expose to morphine or lutein had no effect.

## 4 Discussion

Based on main findings of the current study, prenatal exposure to morphine negatively impacted on reflexive motor behaviors in mice offspring. Morphine exposed in males had no effect on reflexive motor behaviors in mice offspring. No significant difference was seen between male morphine-exposed group compared to male and female morphine exposed group. These results suggested morphine withdrawal syndrome had no effect on reflexive motor behaviors in mice offspring. Paternal, maternal, and parental exposure to morphine during adolescence has been observed to elicit an augmentation in anxiety- and depressive-like behavior in the progeny [15]. Moreover, it has been ascertained that the administration of morphine by the father can induce modifications in diverse physiological processes, encompassing the perception of pain. Additionally, the exposure of the offspring to drug usage by the parents has been demonstrated to exert an adverse influence on memory [22]. In a recent study by Akbarabadi et al. [7], the effects of morphine exposure in parents (either the mother, father, or both) before mating were extensively investigated. The findings of this investigation indicate that parental exposure to morphine before gestation can ameliorate passive avoidance memory and our finding was in agreement to previous reports.

Battery reflexes, a crucial method for assessing neurodevelopmental changes in young animals, includes several tests such as limb grasping and placing, cliff avoidance, righting, accelerated righting, gait, auditory startle, posture, and eye opening [16]. Variations from typical reflexes may lead to delays in acquiring skills and act as a forecasting sign of developmental disorders. Rodents are an excellent model for studying motor skills by using battery reflexes to detect neurodevelopmental disorders and reflex delays [17]. Exposure to morphine during pregnancy is linked to a variety of structural and functional issues, including changes in dendritic length, synaptic plasticity, neuronal growth, and cholinergic function [18]. According to the primary result of this research, no negative impact was observed on reflexive motor activities in the offspring of male mice that were administered morphine followed by lutein. Exposure to lutein during pregnancy had a protective effect on reflexive motor behaviors in the offspring of mice experiencing morphine withdrawal syndrome. No notable difference was observed between males and females treated with morphine when compared to females exposed to both morphine and lutein.

Lutein administration decreased serum MDA production and decreased SOD, GPx and TAS levels in offspring of the female morphine exposed mice. The administration of nanoparticles containing lutein (5 mg/kg) effectively ameliorated the deficits in sociability, social memory, and anxiety-like and repetitive behaviors that were induced by valproic acid. Moreover, it also restored the markers of oxidative stress and apoptosis in the hippocampus. This pharmacological impact can be attributed to the reversal of autism spectrum disorder-like behaviors. These findings provide compelling evidence for the potential of lutein-loaded nanoparticles as an alternative therapeutic approach for mitigating the behavioral impairments caused by valproic acid in female rats, and suggest a potential role of oxidative stress in this process [19]. The complex molecular mechanisms that contribute to lutein's protective effects against morphine withdrawal-related impairments in reflexive motor behavior still require thorough examination. The present study is the first to document lutein's protective effect against defects in reflexive motor behavior caused by morphine withdrawal, and we could not compare our findings to any previous reports. It is likely that lutein has a protective effect against oxygen stress. This regulation occurs by reducing the hypoxic trigger that initiates abnormal neovascularization [20].

Lutein treatment downregulates the expression of vascular endothelial growth factor while concurrently augmenting the levels of antioxidant enzymes for age-related macular degeneration autism spectrum disorder -like retinal degeneration, as well as the streptozotocin-induced diabetic retinopathy in mouse model [21]. It seems, maternal exposure to lutein had protective role on adverse effect of the morphine withdrawal on reflexive motor behaviors in offspring and these effects might related to its antioxidant properties. Nonetheless, due to the limitations of this study, we could not perform histopathological evaluations to verify the findings' accuracy. Additional studies are required to elucidate the cellular and molecular mechanisms behind the protective role of lutein against morphine withdrawal syndrome on reflexive motor behavior in mouse offspring.

## CRediT authorship contribution statement

**Sahand Sariaslani:** Writing – original draft, Conceptualization. **Shahin Hassanpour:** Writing – review & editing, Supervision, Methodology. **Bita Vazir:** Project administration.

## Disclosure statement

No potential conflict of interest was reported by the authors.

## Declaration of competing interest

The authors declare that they have no known competing financial interests or personal relationships that could have appeared to influence the work reported in this paper.

## References

[1] Liew S.M., Chowdhury E.K., Ernst M.E., Gilmartin‐Thomas J., Reid C.M., Tonkin A., Neumann J., McNeil J.J., Kaye D.M. (2022 Dec). Prescribed opioid use is associated with adverse cardiovascular outcomes in community‐dwelling older persons. ESC heart failure.

[2] Umberger W., Gaddis L. (2020 Feb 1). The science of addiction through the lens of opioid treatment for chronic noncancer pain. Pain Manag. Nurs..

[3] Jiang C., Yang X., He G., Wang F., Wang Z., Xu W., Mao Y., Ma L., Wang F. (2021 Nov). CRHCeA→ VTA inputs inhibit the positive ensembles to induce negative effect of opiate withdrawal. Mol. Psychiatr..

[4] Luzzati M., Coviello C., De Veye H.S., Dudink J., Lammertink F., Dani C., Koopmans C., Benders M., Tataranno M.L. (2023 Jan 17). Morphine exposure and neurodevelopmental outcome in infants born extremely preterm. Dev. Med. Child Neurol..

[5] Smith B.L., Guzman T.A., Brendle A.H., Laaker C.J., Ford A., Hiltz A.R., Zhao J., Setchell K.D., Reyes T.M. (2022 Sep 1). Perinatal morphine exposure leads to sex-dependent executive function deficits and microglial changes in mice. eneuro.

[6] Jantzie L.L., Maxwell J.R., Newville J.C., Yellowhair T.R., Kitase Y., Madurai N., Ramachandra S., Bakhireva L.N., Northington F.J., Gerner G., Tekes A. (2020 Feb 1). Prenatal opioid exposure: the next neonatal neuroinflammatory disease. Brain Behav. Immun..

[7] Akbarabadi A., Sadat-Shirazi M.S., Kabbaj M., Zadeh-Tehrani S.N., Khalifeh S., Pirri F., Zarrindast M.R. (2021 Jul 1). Effects of morphine and maternal care on behaviors and protein expression of male offspring. Neuroscience.

[8] Bardo M.T., Hammerslag L.R., Malone S.G. (2021 Jun 15). Effect of early life social adversity on drug abuse vulnerability: focus on corticotropin-releasing factor and oxytocin. Neuropharmacology.

[9] Park H.A., Hayden M.M., Bannerman S., Jansen J., Crowe-White K.M. (2020 Jul 29). Anti-apoptotic effects of carotenoids in neurodegeneration. Molecules.

[10] Dewanjee S., Zia-Ul-Haq M., Riaz M., Sarkhel S., Chakraborty P., Ahmed S. (2021).

[11] Rezai M., Mahmoodi M., Kaeidi A., Karimabad M.N., Khoshdel A., Hajizadeh M.R. (2018 Aug 1). Effect of crocin carotenoid on BDNF and CREB gene expression in brain ventral tegmental area of morphine treated rats. Asian Pac. J. Trop. Biomed..

[12] Farkhondeh T., Samarghandian S., Yazdi H.S., Samini F. (2018). The protective effects of crocin in the management of neurodegenerative diseases: a review. American journal of neurodegenerative disease.

[13] Feather-Schussler D.N., Ferguson T.S. (2016). A battery of motor tests in a neonatal mouse model of cerebral palsy. JoVE J..

[14] Heyser C.J. (2003 Oct). Assessment of developmental milestones in rodents. Current protocols in neuroscience.

[15] Odegaard K.E., Pendyala G., Yelamanchili S.V. (2021 Mar). Generational effects of opioid exposure. Encyclopedia.

[16] Nguyen A.T., Armstrong E.A., Yager J.Y. (2017). Neurodevelopmental reflex testing in neonatal rat pups. JoVE J..

[17] Semple B.D., Blomgren K., Gimlin K., Ferriero D.M. (2013). Noble-Haeusslein LJ Brain development in rodents and humans: identifying benchmarks of maturation and vulnerability to injury across species. Prog. Neurobiol..

[18] Li Z., Santhanam P., Coles C.D., Ellen Lynch M., Hamann S., Peltier S. (2013). Prenatal cocaine exposure alters functional activation in the ventral prefrontal cortex and its structural connectivity with the amygdala. Psychiatry Res..

[19] Viana C.E., Bortolotto V.C., Araujo S.M., Dahleh M.M., Machado F.R., de Souza Pereira A., de Oliveira B.P., Leimann F.V., Gonçalves O.H., Prigol M., Guerra G.P. (2023 Jan 1). Lutein-loaded nanoparticles reverse oxidative stress, apoptosis, and autism spectrum disorder-like behaviors induced by prenatal valproic acid exposure in female rats. Neurotoxicology.

[20] Arunkumar R., Li B., Addo E.K., Hartnett M.E., Bernstein P.S. (2023 Apr 3). Prenatal carotenoid supplementation with lutein or zeaxanthin ameliorates oxygen-induced retinopathy (OIR) in Bco2−/− macular pigment mice. Investig. Ophthalmol. Vis. Sci..

[21] Fu Z., Meng S.S., Burnim S.B., Smith L.E., Lo A.C. (2017 Jul). Lutein facilitates physiological revascularization in a mouse model of retinopathy of prematurity. Clin. Exp. Ophthalmol..

[22] Ahmadalipour A., Rashidy-Pour A. (2015 Feb 1). Effects of treadmill running exercise during the adolescent period of life on behavioral deficits in juvenile rats induced by prenatal morphine exposure. Physiol. Behav..

[23] Hosseinzadeh H., Jahanian Z. (2010 May). Effect of Crocus sativus L.(saffron) stigma and its constituents, crocin and safranal, on morphine withdrawal syndrome in mice. Phytother Res.: An International Journal Devoted to Pharmacological and Toxicological Evaluation of Natural Product Derivatives.

[24] Wu A.G., Yong Y.Y., Pan Y.R., Zhang L., Wu J.M., Zhang Y., Tang Y., Wei J., Yu L., Law B.Y., Yu C.L. (2022 Apr 4). Targeting Nrf2-mediated oxidative stress response in traumatic brain injury: therapeutic perspectives of phytochemicals. Oxid. Med. Cell. Longev..

